# Chemogenetic activation of histamine neurons promotes retrieval of apparently lost memories

**DOI:** 10.1186/s13041-024-01111-8

**Published:** 2024-06-15

**Authors:** Yuto Yokoi, Ayame Kubo, Kyoka Nishimura, Yuki Takamura, Yoshikazu Morishita, Masabumi Minami, Hiroshi Nomura

**Affiliations:** 1 Endowed Department of Cognitive Function and Pathology, Institute of Brain Science, Graduate School of Medical Sciences, Nagoya City University, Nagoya, 467-8601 Japan; 2 Department of Pharmacology, Graduate School of Pharmaceutical Sciences, Hokkaido University, Sapporo, 060-0812 Japan

**Keywords:** Histamine, Tuberomammillary nucleus, Memory, Retrieval, Chemogenetics, Perirhinal cortex

## Abstract

Memory retrieval can become difficult over time, but it is important to note that memories that appear to be forgotten might still be stored in the brain, as shown by their occasional spontaneous retrieval. Histamine in the central nervous system is a promising target for facilitating the recovery of memory retrieval. Our previous study demonstrated that histamine H3 receptor (H3R) inverse agonists/antagonists, activating histamine synthesis and release, enhance activity in the perirhinal cortex and help in retrieving forgotten long-term object recognition memories. However, it is unclear whether enhancing histaminergic activity alone is enough for the recovery of memory retrieval, considering that H3Rs are also located in other neuron types and affect the release of multiple neurotransmitters. In this study, we employed a chemogenetic method to determine whether specifically activating histamine neurons in the tuberomammillary nucleus facilitates memory retrieval. In the novel object recognition test, control mice did not show a preference for objects based on memory 1 week after training, but chemogenetic activation of histamine neurons before testing improved memory retrieval. This selective activation did not affect the locomotor activity or anxiety-related behavior. Administering an H2R antagonist directly into the perirhinal cortex inhibited the recovery of memory retrieval induced by the activation of histamine neurons. Furthermore, we utilized the Barnes maze test to investigate whether chemogenetic activation of histamine neurons influences the retrieval of forgotten spatial memories. Control mice explored all the holes in the maze equally 1 week after training, whereas mice with chemogenetically activated histamine neurons spent more time around the target hole. These findings indicate that chemogenetic activation of histamine neurons in the tuberomammillary nucleus can promote retrieval of seemingly forgotten object recognition and spatial memories.

## Introduction

Memory retrieval can become challenging over time, a process often accelerated by various neurological and psychiatric disorders [1–3], negatively impacting quality of life. However, even memories that seem forgotten may be still stored latently in the brain, as evidenced by their occasional spontaneous recollection. Thus, enhancing positive modulators for memory retrieval might help recover these seemingly lost memories. While some studies have reported retrieval recovery in animals and humans [4–6], the underlying mechanisms remain largely unexplored.

Histamine in the central nervous system represents a potential target for restoring memory retrieval [7]. Brain histamine is produced mainly in tuberomammillary nucleus (TMN) neurons, is released across various brain regions, and plays a role in learning and memory, wakefulness, motivation, and energy balance [8–10]. Histamine H3 receptor (H3R) inverse agonists/antagonists stimulate the histaminergic nervous system by increasing histamine synthesis and release [11]. We have previously shown that H3R inverse agonists/antagonists enhance perirhinal cortex (PRh) activity and restore the retrieval of forgotten long-term object recognition memories in mice [4]. Similar effects on recognition memory retrieval have been observed in humans [4]. Other studies have also reported that H3R inverse agonists/antagonists enhance memory retrieval [12, 13]. However, it remains unclear whether heightened histaminergic activity alone is sufficient for restoring memory retrieval, as H3Rs are located in other neuron types and influence the release of various transmitters (e.g., γ-aminobutyric acid (GABA), glutamate, acetylcholine, and noradrenaline) [14–16]. Indeed, H3R inverse agonists/antagonists can affect brain functions through modulation of dopamine [17].

In this study, we selectively activated histamine neurons using a chemogenetic approach [18] to determine whether this selective activation is sufficient to induce memory retrieval recovery. We also investigated the necessity of activating histamine H2 receptors in this process. Finally, we employed the Barnes maze test to assess the effectiveness of chemogenetic activation of histamine neurons in retrieving spatial memories.

## Results

**Fig. 1:**
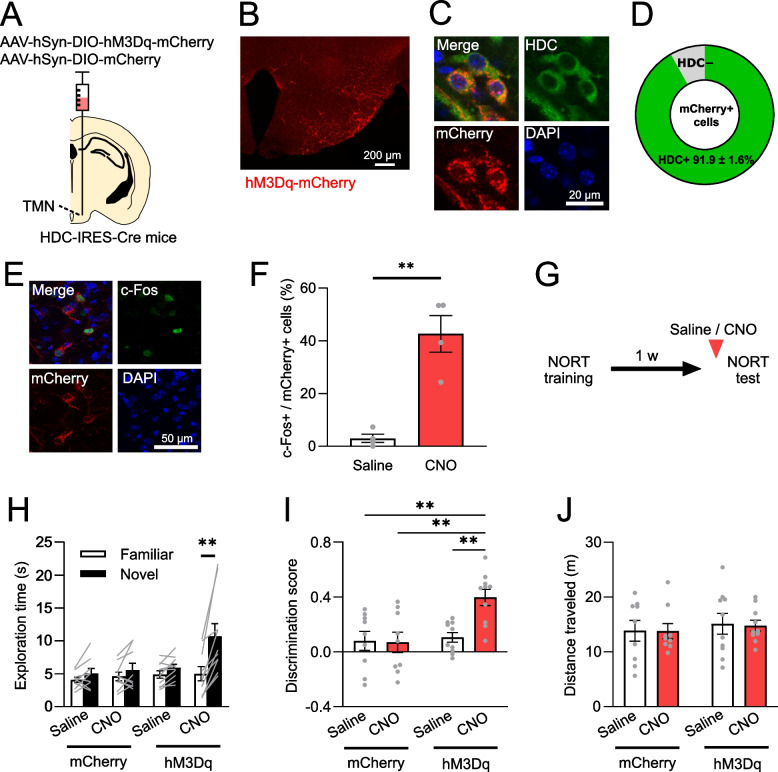
Enhanced retrieval of object recognition memories via chemogenetic activation of histamine neurons. **A** Illustration depicting the injection of AAV8-hSyn-DIO-hM3Dq-mCherry or AAV8- hSyn-DIO-mCherry into the TMN of HDC-IRES-Cre mice. **B** Confocal microscopy image showing hM3Dq-mCherry expression in the TMN. **C** Representative images showing HDC expression in hM3Dq-mCherry positive neurons. **D** The proportion of HDC positive neurons among hM3Dq-mCherry positive neurons. *N* = 6 mice. **E** Representative images showing c-Fos expression in hM3Dq-mCherry positive neurons. **F** Administration of CNO increased the proportion of c-Fos positive neurons among hM3Dq-mCherry positive neurons (**P = 0.0014, unpaired t-test). hM3Dq-Saline: *N* = 4 mice, hM3Dq-CNO: *N* = 4 mice. **G** Novel object recognition memory was assessed 1-week post-training. **H** Mice with hM3Dq in histamine neurons treated with CNO showed a preference for exploring the novel object (**P < 0.0001, Sidak’s test after two-way repeated measures ANOVA (interaction, F(3, 34) = 8.60, *P* = 0.0002)). **I** The discrimination score, a measure of distinguishing novel objects from familiar objects, was greater in the hM3Dq-CNO group compared to the control groups (**P < 0.01, Tukey’s test after two-way ANOVA (interaction, F(1, 34) = 6.35, *P* = 0.0166)). **J** The distance traveled during the test session was comparable across groups. mCherry-Saline: *N* = 9 mice, mCherry-CNO: *N* = 9 mice, hM3Dq-Saline: *N* = 10 mice, hM3Dq-CNO: *N* = 10 mice. Values are reported as mean ± SEM

To virally target hM3Dq, the Gq-coupled excitatory designer receptor exclusively activated by designer drugs (DREADDs), to histamine neurons, we infused an adeno-associated virus (AAV) 8-hSyn-DIO-hM3Dq-mCherry into the TMN of histidine decarboxylase (Hdc)-IRES-Cre mice (hM3Dq mice) (Fig. 1A, B). We verified that 91.9 ± 1.6% of the hM3Dq-mCherry + neurons were positive for Hdc (Fig. 1C, D). To confirm whether an intraperitoneal injection of clozapine-N-oxide (CNO) induces activation of histamine neurons, we performed an immunohistochemical analysis of c-Fos. The CNO injection increased c-Fos expression in the hM3Dq-mCherry + neurons (Fig. 1E, F).

**Fig. 2:**
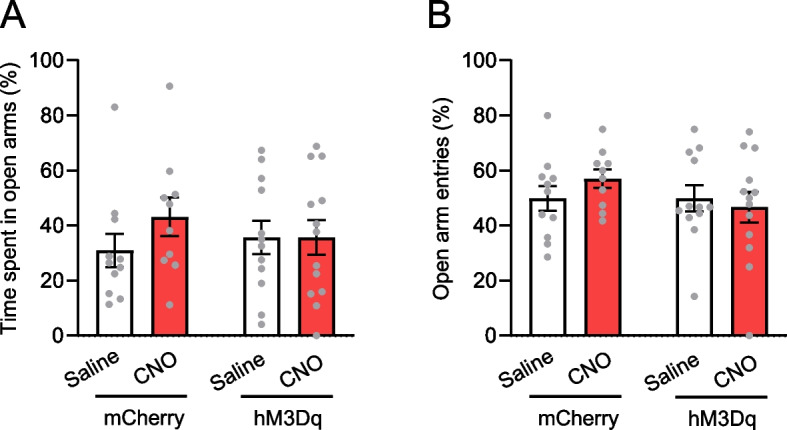
No impact of chemogenetic activation of histamine neurons on anxiety-related behavior. **A** Time spent in open arms of the elevated plus maze was consistent across the 4 behavioral groups. **B** The frequency of visits to open arms was similar across all groups. mCherry-Saline: *N* = 11 mice, mCherry-CNO: *N* = 10 mice, hM3Dq-Saline: *N* = 12 mice, hM3Dq-CNO: *N* = 13 mice. Values are reported as mean ± SEM

We utilized the novel object recognition test as a measure of memory, which examines whether the mouse can distinguish between novel objects and objects previously encountered during the training session. Our previous study indicated that mice fail to discriminate novel objects from familiar objects 3 days after training [4]. However, administering thioperamide, an H3R inverse agonist/antagonist, during the test period between 3 days and 1 month after training restored this ability to discriminate. Therefore, in this study, the test session was conducted 1 week after the training session (Fig. 1G). When the hM3Dq mice were given an intraperitoneal injection of CNO during the test session, they showed increased exploration of the novel object compared with the familiar object (Fig. 1H). For control groups, we had mice with hM3Dq-mCherry in histamine neurons given saline and mice with mCherry in histamine neurons given either saline or CNO. These control groups did not show a preference for the novel object. To quantify this effect, we calculated the discrimination score, reflecting the ability to discriminate between novel and familiar objects. The score for the hM3Dq-CNO group was greater than those of the control groups (Fig. 1I). Additionally, the distance traveled during the test session was comparable across all 4 groups (Fig. 1J). Furthermore, we evaluated anxiety-related behavior using the elevated plus maze test, as anxiety levels might affect memory retrieval. The time spent in open arms and the number of visits to open arms were consistent across all groups (Fig. 2). These results indicate that chemogenetic activation of histamine neurons promotes the retrieval of forgotten object memories.

**Fig. 3:**
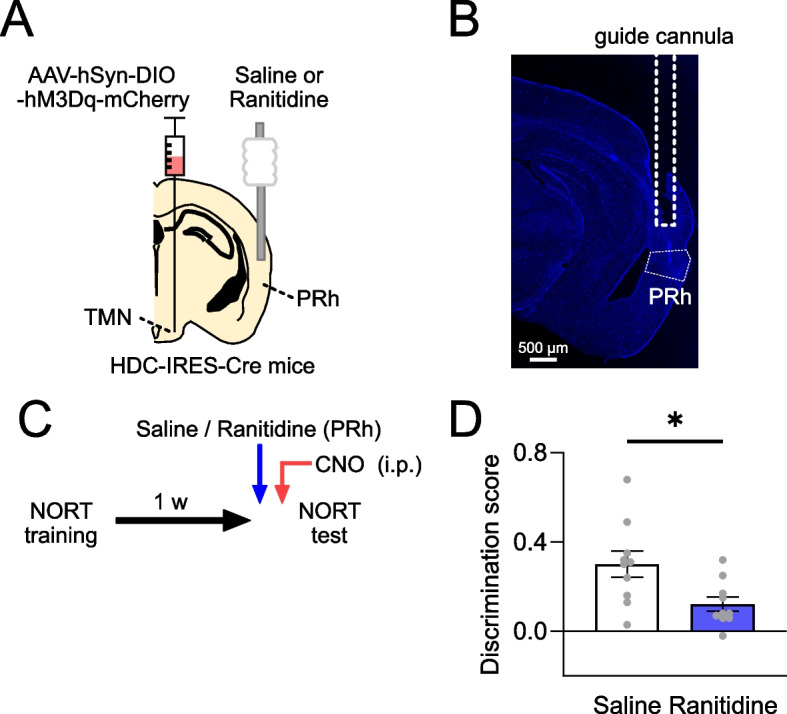
Activation of histamine H2 receptors in the PRh is necessary for the retrieval recovery. **A** Illustration depicting the placement of a guide cannula above the PRh. **B** A representative image showing the trajectory of the guide cannula, positioned 1 mm above the PRh. **C** Mice with hM3Dq expressed in histamine neurons were subjected to an object recognition memory test 1 week after their training session. Thirty minutes before the test, they received either ranitidine or saline directly into the PRh, followed by administration of CNO. **D** Mice treated with ranitidine showed a reduced discrimination score in comparison to those treated with saline. *P = 0.0153, unpaired t-test. *N* = 10 mice. Values are reported as mean ± SEM

We investigated whether the activation of histamine receptors plays a role in the recovery of memory retrieval. The PRh is a critical region for the novel object recognition test. The H2 receptor (H2R) is expressed in the PRh [19], and our previous study indicated that H2R activation in the PRh is critical for improving the retrieval of forgotten object recognition memories by H3R inverse agonists/antagonists [4]. Therefore, we aimed to determine whether H2R activation in the PRh is necessary for memory retrieval prompted by chemogenetic activation of histamine neurons. Mice with hM3Dq in their histamine neurons were given a local administration of either ranitidine, an H2R antagonist, or saline via infusion cannulas in the PRh 30 min before the test session (Fig. 3A-C). This was followed by intraperitoneal administration of CNO to all the mice. Mice treated with saline exhibited a preference for exploring the novel object, consistent with previous findings (Fig. 1D). In contrast, mice treated with ranitidine showed reduced exploration of the novel object, similar to their interaction with the familiar object (Fig. 3D). The discrimination score was lower in the mice administered ranitidine compared to those receiving saline. These results suggest that H2R activation in the PRh is required for the successful recovery of memory retrieval.

**Fig. 4:**
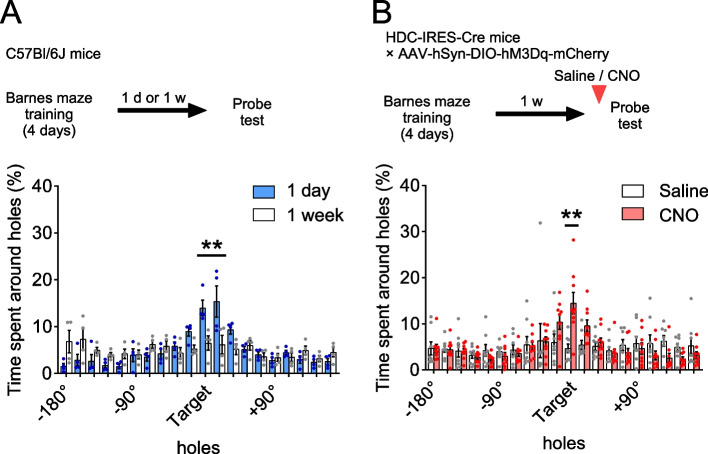
Chemogenetic activation of histamine neurons promotes retrieval of forgotten spatial memories. **A** Mice underwent 4 days of training in the Barnes maze. Memory testing occurred either 1 day or 1 week after the last training day. The mice showed a preference for the target and adjacent holes in the 1-day test, but no hole preference was observed in the 1-week test (**P < 0.001, Sidak’s test after two-way repeated measures ANOVA (interaction, F(19, 114) = 4.53, *P* < 0.0001)). *N* = 4 mice. **B** Mice with hM3Dq underwent the same training and a probe test 1 week later. The mice with CNO spent more time around the target hole compared to those treated with saline (**P < 0.0001, Sidak’s test after two-way repeated measures ANOVA (interaction, F(19, 285) = 2.87, *P* < 0.0001)). Saline: *N* = 8 mice, CNO: *N* = 9 mice. Values are reported as mean ± SEM

Finally, we examined whether chemogenetic activation of histamine neurons influences the retrieval of forgotten spatial memories using the Barnes maze test. Over 4 days of training, mice learned which hole on the platform had a box underneath them to escape. The probe test, conducted either 1 day or 1 week after the training with the escape box removed, assessed their memory retention. Results from the one-day test showed that mice spent more time around the hole where the escape box was originally located and its adjacent holes (Fig. 4A). On the other hand, in the 1-week test, the mice did not show a preference for any specific hole. Based on these results, we chose a 1-week interval between training and testing for subsequent experiments.

The mice with hM3Dq-mCherry in their histamine neurons underwent 4 days of Barnes maze training. One week after the final training session, they were subjected to the probe test. Either saline or CNO was administered 30 min before this test. The mice given CNO spent more time at the target hole compared to those treated with saline (Fig. 4B). These findings indicate that chemogenetic activation of histamine neurons promotes the retrieval of forgotten spatial memories.

## Discussion

In our study, we employed a chemogenetic method to selectively activate histamine neurons and found that this selective activation alone is sufficient to restore the retrieval of object recognition memories. It is important to note that the novel object recognition test can be influenced by various internal states, such as anxiety and activity levels. However, our findings indicate that activating histamine neurons did not affect anxiety-related behavior or locomotor activity in these mice. Furthermore, we discovered that administering an H2R antagonist directly into the PRh hindered the recovery of memory retrieval. Given the role of the PRh in processing object recognition memories, it is likely that the activation of histamine neurons positively affects the PRh neuronal activity underlying memory traces through H2R activation, thereby facilitating the recovery of memory retrieval.

The improved memory retrieval observed in our study may be attributed to the excitatory effects of histamine through H2R activation [7]. H2R activation leads to an increase in intracellular cyclic AMP (cAMP) and activation of protein kinase A(PKA). This sequence of events reduces afterhyperpolarization by suppressing Ca^2+^-dependent K^+^ channels, thereby increasing neuronal excitability. Additionally, cAMP directly influences the hyperpolarization-activated cation channel HCN2, causing depolarization [20]. Moreover, H2R activation also diminishes the activity of inhibitory interneurons via K_v3.2_-containing K^+^ channels, further influencing the activity within neural networks [21]. These excitatory impacts of histamine may play a role in enhancing memory retrieval. In fact, inducing depolarization in neurons in the PRh replicates the recovery of memory retrieval that are caused by injections of H3R inverse agonist/antagonist injections [4] or the chemogenetic activation of histamine neurons. Given that the reactivation of neurons integrated into memory traces and the synchronized neuronal activity are crucial for memory retrieval [22–27], histamine could facilitate this reactivation and/or synchronized activity for enhanced retrieval. Indeed, histamine has been shown to increase the reactivation of behavior-relevant PRh neuronal populations in brain slices [4], and an H3R inverse agonist/antagonist enhances synchronized activity in the PRh in vivo [28]. However, further studies employing in vivo neuronal recordings [29] are needed for a more precise understanding of how histamine induces the recovery of memory retrieval.

Determining whether the enhancement of memory retrieval through histamine neuron activation is a common feature across various memory tasks is crucial for identifying the mechanisms underlying retrieval enhancement. Previous studies have explored the relationship between pharmacological activation of the histamine system and memory retrieval. However, these studies were limited to specific memory tasks, such as object and social recognition memory tasks, and inhibitory avoidance [12, 13, 30]. Focusing on a single memory task makes it challenging to eliminate confounding factors and to determine whether an intervention modulates a neural basis specific to that memory task or common to memory retrieval. Therefore, in this study, we utilized the Barnes maze test, a method not previously employed in studies of histamine-induced retrieval enhancement, to test whether the activation of histamine neurons facilitates the retrieval of spatial memories. Our findings suggest that histamine does not act on processes specific to object recognition memory but may activate a common neural foundation for memory retrieval. Considering the potential involvement of distinct neural circuits in spatial and novel object recognition memories [31], further research is needed to clarify the significance of the H2R signaling in boosting spatial memory retrieval.

Our study shows that activating histamine neurons does not affect locomotor activity during the test session of the novel object recognition test. This finding contrasts with a previous study that observed an increase in locomotor activity following the same manipulation [32]. This discrepancy could stem from differences in the behavioral task and the timing of experiments. We presented objects to mice to assess memory, and the test was performed during the light-on phase, whereas Yu et al. performed the test in an object-free open field during the light-off phase [32].

Although our results indicate that histamine neuron activation does not influence anxiety-related behavior, it is important to note that this does not rule out a potential association between histamine and anxiety. Previous studies demonstrated that histamine may be involved in anxiety. The lesion of TMN decreases anxiety-related behavior [33], while elevating histamine levels through thioperamide increases anxiety-related behavior only when pretreated with zolantidine, an H2R antagonist [34]. In addition, mice lacking Hdc exhibit more anxiety-related behavior [35]. These findings suggest a complex role of histamine in modulating anxiety.

In conclusion, we demonstrated that chemogenetic activation of histamine neurons promotes the retrieval of object recognition and spatial memories. Future circuit and molecular analyses will determine the mechanisms underlying the histamine-mediated recovery of memory retrieval.

## Methods

### Animals

Adult male mice, aged 7–12 weeks, including histidine decarboxylase-IRES-Cre (HDC-IRES-Cre, #021198, Jackson Laboratory) [36] and C57Bl/6 J mice (Japan SLC, Hamamatsu, Japan) were housed individually. They were kept under a 12-h light–dark cycle, with lights turning on at 07:00 AM, and had unrestricted access to food and water. Behavior tests were conducted during the light phase of this cycle. Animal experiments were performed with the approval of the Institutional Animal Care and Use Committee of Hokkaido University (approval number: 16–0043) and Nagoya City University (approval number: 22–018). The study adhered to the Hokkaido University and Nagoya City University guidelines for the care and use of laboratory animals and complied with several national guidelines: the Guidelines for Proper Conduct of Animal Experiments (Science Council of Japan), the Fundamental Guidelines for Proper Conduct of Animal Experiments and Related Activities in Academic Research Institutions (Ministry of Education, Culture, Sports, Science and Technology, Notice No. 71 of 2006) and, the Standards for Breeding and Housing of and Pain Alleviation for Experimental Animals (Ministry of the Environment, Notice No. 88 of 2006).

### Drugs

CNO (Enzo Life Sciences) was prepared in a solution of 0.5% DMSO in saline and administered to the mice via intraperitoneal injection at a dose of 0.01 ml/g body weight. The control treatment consisted of an identical volume of 0.5% DMSO in saline. The chosen CNO dose (1 mg/kg) was based on prior studies [37]. Ranitidine hydrochloride (Tokyo Chemical Industry, Tokyo, Japan) was dissolved in saline and directly administered into the PRh. The control group received a comparable volume of saline. The dose of ranitidine was selected based on our previous study [4].

### Surgery

The viral vectors pAAV-hSyn-DIO-hM3D(Gq)-mCherry and pAAV-hSyn-DIO-mCherry, kindly provided by Bryan Roth (Addgene viral prep # 44361-AAV8, 50459-AAV8) [38] were used in the study. Mice were anesthetized with isoflurane (0.8–1.5%) and secured in a stereotaxic frame (SR-6 M-HT, Narishige, Tokyo, Japan). To minimize pain, lidocaine (2%; Aspen Japan, Tokyo, Japan) was applied topically to the scalp. We injected AAV8-hSyn-DIO-hM3D(Gq)-mCherry (2.2 × 10^13^ virus molecules/mL, 0.5 µL) or AAV8-hSyn-DIO-mCherry (4.1 × 10^13^ virus molecules/mL, 0.5 µL) into the bilateral TMN (A/P: -2.5 mm, M/L: ± 0.92 mm, D/V: -5.34 mm) at a rate of 0.1 µL/min. After injection, the infusion cannulas (33 gauge) were left in place for at least 10 min to ensure effective distribution of the solution. Behavioral tests were carried out after a waiting period of at least 3 weeks, allowing time for the expression of the transgenes in the mice.

To infuse the H2R antagonist into the PRh, guide cannulas were implanted bilaterally 1 mm above the PRh (A/P: -3.05 mm, M/L: ± 4.55 mm, D/V: -2.8 mm) and secured with a self-curing adhesive resin cement (Super-Bond, SUN MEDICAL, Moriyama, Japan). Dummy cannulas (33-gauge) were then inserted into each guide cannula to prevent clogging. Mice were given at least 7 days for postoperative recovery.

### Novel object recognition test

The test was conducted similarly to that in our previous study [4] with minor modifications. Mice first underwent habituation sessions for three consecutive days, during which they explored an open field (32 cm × 32 cm × 35 cm) for 15 min each day. During the training session, they were placed in the field with two identical objects and allowed to explore for 15 min. In the test session, they explored for 5 min in the presence of one familiar object and one novel object. These objects were similar in texture and size but distinct in shape. The roles of familiar and novel objects were counterbalanced among the mice. A discrimination score was calculated for each mouse as the ratio (T2-T1)/(T1 + T2) [T1 = time spent exploring the familiar object, T2 = time spent exploring the novel object]. The test area and objects were cleaned with 70% ethanol solution between trials. All sessions were recorded by a camera, and the video was analyzed by using either Noldus Ethovision XT 10 software (Fig. 1) or DeepLabCut [39] (Fig. 3). Exploration was defined as the mouse’s nose being within 4 cm of an object’s center, excluding sitting on the object.

### Elevated plus maze test

The test was conducted similarly to that in our previous study [40] with minor modifications. Mice were positioned at the center of the elevated plus maze, which consisted of a central area (8 cm by 8 cm) and four extending arms. Two of these arms were open, each measuring 8 cm wide and 25 cm long, while the other two were enclosed, having the same dimensions but with 25 cm-high walls on the sides and end. At the start of each test, the mice were placed in the central section, facing one of the enclosed arms. The animals’ movements were tracked over 5 min using a camera fixed above the maze’s center. The duration the animal spent in the open and closed arms, and the number of entries into the arms were calculated using Noldus Ethovision XT 10 software. An entry into any arm was considered valid when the animal placed all four paws into that arm.

### Barnes maze test

The Barnes maze test was conducted using a circular platform, which was brightly lit at 360–390 lx and 90 cm in diameter, and elevated 76 cm above the floor. This platform featured 20 holes, each 4.5 cm in diameter, positioned 5 cm from its edge. On the first day, the mice were familiarized with an escape box measuring 15 × 9 × 6 cm for 3 min. From the second to the fifth day, they underwent training sessions. During these sessions, the platform had an escape box placed beneath one of the holes. The location of the escape box was fixed across 4 days of training and different among the mice. The mice, initially placed at the platform’s center and covered by a holding chamber, were given 10 s before the chamber was removed. They then had 180 s to freely explore and find the escape box. The session concluded when a mouse fully entered the escape box. These sessions were conducted thrice daily, at 20-min intervals. On either the sixth or twelfth day, a probe test was carried out, during which the escape box was removed from the platform. The procedure mirrored the training sessions but lasted only 90 s. The mice’s behavior was captured by a camera, and the amount of time spent within 5 cm from the center of each hole was analyzed using Noldus Ethovision XT 10 software.

### Microinfusions

For the microinfusions, 0.5 µL of the solution was administered to each side using 28-gauge infusion cannulas. These cannulas extended 1 mm below the guide cannulas and were operated with a pump for 2 min. To ensure effective diffusion of the solutions, the infusion cannulas remained in place for at least 2 min following the infusion.

### Histology

After the behavioral experiments, the mice were deeply anesthetized using either pentobarbital or an anesthetic mixture of medetomidine (0.75 mg/kg), midazolam (4.0 mg/kg), and butorphanol (5.0 mg/kg). They were then transcardially perfused with phosphate-buffered saline (PBS), followed by 4% paraformaldehyde (PFA) solution. The brains were post-fixed in 4% PFA overnight at 4 °C, then cryoprotected in 15% and 30% sucrose solutions dissolved in PBS at 4 °C for 48–72 h. Coronal slices, each 40 µm thick, were prepared using a cryostat (CM3050S, Leica). Slices were mounted on slides and stained for nuclei with 4′,6-diamino-2-phenylidole (DAPI) (0.3 µg/ml, 4′,6-diamidino-2-phenylindole, Cat. #340–07971, Dojindo Laboratories, Kumamoto, Japan). To confirm the exact locations of mCherry expression and cannula implantation, images of these slices were captured using a fluorescence microscope (BZ-X700, Keyence) or confocal microscope system (A1RS + , Nikon) at 10 × magnification.

### Immunohistochemistry

Immunohistochemistry was carried out following tissue processing. Tissue sections were incubated in PBST (0.1% Triton X-100 in PBS) for 15 min at room temperature (RT). For HDC immunostaining, they were then blocked using PBS-BX (3% BSA, 10.25% TritonX-100 in PBS) for 1 h at RT. The sections were incubated with a rabbit polyclonal antibody against HDC (dilution 1:800, POG, Cat. #16045) overnight at 4 °C. Following antibody incubation, the sections were washed three times with PBS-BX for 15 min each, then incubated with AlexaFluor 488 Goat anti-rabbit IgG (dilution 1:1000, Invitrogen, Cat. #A32731) for 2 h at RT. This was followed by 5-min staining with DAPI in PBS. For c-Fos immunostaining, the sections were then blocked using PBST containing 10% normal goat serum (Abcam, Cat. #ab7481) for 1 h at RT. They were treated with a rabbit polyclonal antibody against c-Fos (1:1000, Millipore, Cat. #ABE457) overnight at 4 °C. Following antibody incubation, the sections were washed three times with PBST for 15 min each, then incubated with AlexaFluor 488 Goat anti-rabbit IgG for 2 h at RT. This was followed by 5-min staining with DAPI in PBS. After two additional 5-min washes in PBS, the sections were mounted on glass slides with a mounting medium (20 mM Tris, 0.5% N-propyl gallate, 90% glycerol, pH 8.0). Imaging was performed using a laser-scanning confocal microscope (A1RS + , NIKON, Tokyo, Japan). The proportion of HDC or c-Fos positive cells within mCherry positive cells in the TMN was calculated.

### Statistical analyses

Values are reported as mean ± SEM (standard error of the mean). Statistical analysis was performed using two-way analysis of variance (ANOVA), repeated-measures ANOVA, Tukey’s test, Sidak’s test, and two-sided unpaired *t*-test, where appropriate.

## Data Availability

The datasets used and analyzed during the current study are available from the corresponding author upon reasonable request.
